# Cardiac calcium regulation in human induced pluripotent stem cell cardiomyocytes: Implications for disease modeling and maturation

**DOI:** 10.3389/fcell.2022.986107

**Published:** 2023-01-18

**Authors:** Patrick Ernst, Philip A. Bidwell, Michaela Dora, David D. Thomas, Forum Kamdar

**Affiliations:** ^1^ Cardiovascular Division, University of Minnesota, Minneapolis, MN, United States; ^2^ College of Biological Sciences, University of Minnesota, Minneapolis, MN, United States; ^3^ Department of Biochemistry, Molecular Biology, and Biophysics, University of Minnesota, Minneapolis, MN, United States

**Keywords:** calcium, stem cells, cardiomyocytes, disease modeling, maturation, human induced pluripotent stem cells, genetic cardiovascular diseases

## Abstract

Human induced pluripotent stem cell cardiomyocytes (hiPSC-CMs) are based on ground-breaking technology that has significantly impacted cardiovascular research. They provide a renewable source of human cardiomyocytes for a variety of applications including *in vitro* disease modeling and drug toxicity testing. Cardiac calcium regulation plays a critical role in the cardiomyocyte and is often dysregulated in cardiovascular disease. Due to the limited availability of human cardiac tissue, calcium handling and its regulation have most commonly been studied in the context of animal models. hiPSC-CMs can provide unique insights into human physiology and pathophysiology, although a remaining limitation is the relative immaturity of these cells compared to adult cardiomyocytes Therefore, this field is rapidly developing techniques to improve the maturity of hiPSC-CMs, further establishing their place in cardiovascular research. This review briefly covers the basics of cardiomyocyte calcium cycling and hiPSC technology, and will provide a detailed description of our current understanding of calcium in hiPSC-CMs.

## Introduction

Since Ringer’s initial discovery of the critical contribution of extracellular calcium (Ca^2+^) to cardiac contractility in 1833, extensive research using fluorescent calcium indicators has delineated the ever-expanding role of Ca^2+^ in muscle, and the heart in particular, beyond the second messenger controlling contraction to a regulator of critical processes such as energetics and cell survival in healthy and diseased hearts. (Ringer, 1883). e. Aberrant Ca^2+^ homeostasis is a nearly universal characteristic of both acquired and genetic forms of human heart failure, an observation reinforced by an extensive array of experimental models of disease. Dysregulation of Ca^2+^ handling not only depresses the mechanical function of the heartbut also triggers numerous signaling pathways that contributes to the progression of heart failure and cardiovascular disease.

Our understanding of cardiac Ca^2+^ handling has been built upon extensive experimentation on various large and small animal models, given the exceedingly limited availability of human cardiac tissue. Early efforts emphasized larger animal models (e.g., porcine and canine) that most closely mimic human physiology on a cellular and organ level (Bers, 2008; Eisner et al., 2017). In recent decades, murine models have been at the forefront of preclinical cardiovascular research, with genetic modification (knockout, overexpression, introduction of mutations) has proven invaluable in delineating the role of individual proteins and their function within complex signaling pathways and networks under physiological and pathophysiological conditions. A major caveat of this research is the significant difference in the size and mechanics of the mouse and human hearts.

The limitations of small animal models and heterologous expression systems codify the need to faithfully recapitulate the genetic, epigenetic, and molecular framework of the human system, especially when considering the validity of cardiovascular models. Recent developments in human induced pluripotent stem cell (hiPSC) technology and somatic reprogramming of hiPSCs, using the Yamanaka factors (Oct3/4, Sox2, Klf4, and c-Myc) (Takahashi and Yamanaka, 2006), offer a partial solution. The subsequent capacity to differentiate into cardiomyocytes (hiPSC-CMs) has provided a new avenue for investigation of cardiac cell physiology (Yang et al., 2008). A distinct advantage over previous models is the ability to generate patient-derived hiPSC lines that display the exact genetic architecture of an individual, enabling study of nuances of phenotype variability. Patient-derived hiPSCs have become a primary tool in precision medicine, including drug toxicity screening. While hiPSC-CMs technology overcomes the need to translate research between species to assess the human condition, hiPSC-CMs are also relatively immature, not fully reflecting adult cardiomyocytes, and have limited ability to assess organ and systemic effects of cardiac function and disease (Karakikes et al., 2015a; Kamdar et al., 2015; Karbassi et al., 2020). However, the field is continually making advances in differentiation strategies to drive enhanced maturity of these cells. Above all, further research on cardiac Ca^2+^ handling will require a multifaceted approach, utilizing several model systems, of which hiPSC-CMs will be a major component.

This review briefly details the major mechanisms of cardiac Ca^2+^ handling and provides an overview of the basics and current state of hiPSC-CMs technology, where numerous other reviews provide in-depth detail. This review focuses on the current understanding of Ca^2+^-handling proteins expression and function in hiPSC-CMs, and how this relates to existing knowledge from other model systems. Because this is a nascent and rapidly changing field with little standardization, much of this information is limited and scattered, so direct apples-to-apples comparisons are often difficult.

## Adult cardiomyocyte Ca^2+^ handling system

**FIGURE 1:**
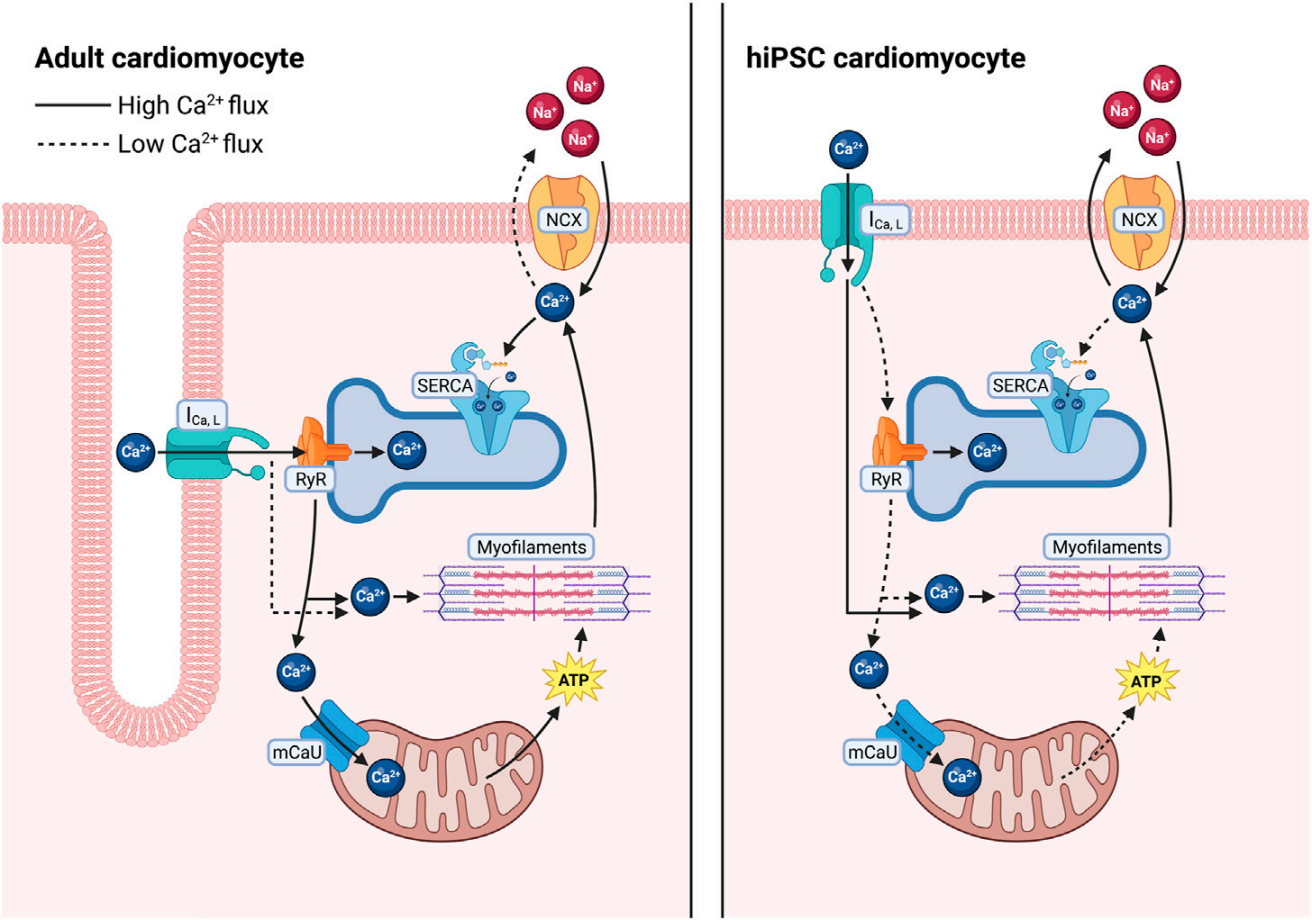
Differences in calcium regulation between adult human cardiomyocytes and in hiPSC-derived cardiomyocytes. In adult human cardiomyocytes (left), T-tubules result in close proximity between L-type Ca^2+^ channels and RyR2 allowing for a larger release of Ca^2+^ from the SR, quickly diffusing into the cytosol and going to the myofilaments to facilitate contraction as well as into closely tethered mitochondria to stimulate ATP production. Ca^2+^ is then primarily sequestered back into the SR *via* SERCA. In hiPSC-CMs (right), increased distance between L-type calcium channels and RyR2 results in less Ca^2+^ release from the SR, requiring the cells to rely more heavily on L-type Ca^2+^ influx to facilitate contraction. After relaxation a larger fraction of the cytosolic Ca^2+^ is removed *via* the Na^+^/Ca^2+^ exchanger.

Cardiomyocyte Ca^2+^ cycling directly mediates cardiac muscle contraction and relaxation. Briefly, sarcolemmal depolarization triggers a Ca^2+^-induced Ca^2+^ release through opening and release of Ca^2+^ through the Cav1.2 subunit of the L-type Ca^2+^ channel (LTCC), which activates the release of Ca^2+^ stores from the sarcoplasmic reticulum (SR) through opening of the ryanodine receptor 2 (RyR2) (Bers, 2001; Setterberg et al., 2021). Elevated cytosolic Ca^2+^ then activates myofibril contraction. Ca^2+^ is then removed from the cytosol through several mechanisms to allow muscle relaxation. The predominant transport occurs through the cardiac sarcoplasmic reticulum calcium ATPase (SERCA2a, which hydrolyzes ATP to pump Ca^2+^ from the cytosol into the SR (Figure 1), which is also necessary for subsequent Ca^2+^ release events through RyR2 (Bers, 2001; Haghighi et al., 2014). Ca^2+^ can also be transported across the plasma membrane by the Na/Ca^2+^ exchanger (NCX) and the plasma membrane Ca^2+^ ATPase (PMCA) (Bers, 2001; Shattock et al., 2015), and into the mitochondria by the mitochondrial Ca^2+^ uniporter (MCU) (Garbincius et al., 2020; Boyman et al., 2021) (Figure 1). In human cardiomyocytes, approximately 70% of Ca^2+^ is removed by SERCA2a, compared to approximately 90% in rodent cells (Bers, 2001), highlighting a fundamental difference in Ca^2+^ cycling that must be accounted for in these extensively used rodent experimental models.

Ca^2+^ cycling is physiologically regulated by the adrenergic system for the fight-or-flight response, through increases in Ca^2+^ release and uptake capabilities. SERCA is inhibited by the 52-aa transmembrane protein phospholamban (PLB), which lowers the binding affinity of Ca^2+^ (Haghighi et al., 2014). Physiologic activation of adrenergic G-protein-coupled receptors elicits downstream activation of protein kinases, protein kinase A (PKA), and calmodulin kinase II (CaMKII), thus phosphorylating PLB at residues Ser16 and/or Thr17 and alleviating inhibition on SERCA (Haghighi et al., 2014). The resulting increased SERCA activity shortens the duration of Ca^2+^ transient for faster relaxation, enabling adequate chamber filling during accelerated beating as a compensatory mechanism. Enhanced SERCA2a activity also elevates SR Ca^2+^ content for greater Ca^2+^ release, yielding faster and stronger myofibril contractions. This elevated Ca^2+^ release is also enhanced by RyR phosphorylation, leading to greater pore opening (Bers, 2014).

In addition to PLB, several other binding partners modulate SERCA2a activity and cardiomyocyte function. HS-1 associated protein X-1 (HAX-1) binds PLB, enhancing its inhibition of SERCA2a, and it (Zhao et al., 2009) is responsible for approximately half of the basal physiological inhibition of SERCA2a by PLB (Bidwell et al., 2018). A recently discovered integral membrane protein, dwarf open reading frame (DWORF), homologous to PLB, probably binds in a similar manner but activates SERCA activity, thereby increasing cardiomyocyte contractility (Nelson et al., 2016; Li A. et al., 2021). SERCA activity and regulation has been predominantly studied in the context of ventricular function, with Ca^2+^ transport less understood in the atrium, although several key differences have been observed. SERCA expression and Ca^2+^ pumping ability are greater in the atrium compared to the ventricle. In the atrium of the cardiomyocyte, PLB levels are lower, but an additional protein regulator, sarcolipin (SLN) is also expressed (Shaikh et al., 2016). SLN is a 31 amino acid protein, which similarly reduces the Ca^2+^ affinity of SERCA, but can also decrease the maximum velocity of enzymatic turnover (Bal and Periasamy, 2020).

Cardiac pathology is commonly associated with the disruption of this finely tuned Ca^2+^ transport apparatus. In chronic heart failure, a state characterized by pathological chronic sympathetic activation, there is reduction in SERCA2a expression and activity, as well as an increase in the inhibitory function of PLB. These alterations in the heart failure state lead to increased intracellular cardiac Ca^2+^ and reduced SR Ca^2+^ stores, and consequently reduced systolic calcium concentrations (Kranias and Hajjar, 2012; Denniss et al., 2020). Subsequently, these changes stemming from the reduction in SERCA2a activity and expression result in abnormal excitation-contraction coupling, activation of stress signaling pathways, and reduced cardiac contractility (Lipskaia et al., 2007; Kawase and Hajjar, 2008; Kranias and Hajjar, 2012; Denniss et al., 2020). RyR phosphorylation is chronically increased in disease, which is associated with arrhythmogenesis due to increased Ca^2+^ leak (Bers, 2014). Thus, addressing changes in calcium regulation, specifically SERCA2a activation, has been a therapeutic interest in heart failure.

In addition, mitochondria play an important role in cardiac function and cardiac Ca^2+^ regulation. In cardiomyocytes, the mitochondria are in close proximity to the Ca^2+^ store of the SR. (Frederick and Shaw, 2007). The high Ca^2+^ concentration microenvironment results in the influx of Ca^2+^ into mitochondria through the MCU. This mitochondrial Ca^2^ influx is crucial as the mitochondrial Ca^2+^ influx stimulates respiration. Furthermore, inhibiting MCU activity in cardiac mitochondria has been associated with a resulting increase in cytosolic Ca^2+^ transient amplitudes (Drago et al., 2012; Boyman et al., 2014). In addition to the MCU, mitochondrial calcium influx is possible through the rapid mode of Ca^2+^ update (RaM). Although RaM allows for more rapid calcium influx, it is inactivated at calcium concentrations well below those seen in active cardiomyocytes and can take well over a minute to recover from inactivation. As a result, the likelihood of its involvement in beat-by-beat cardiac calcium regulation is slim (Buntinas et al., 2001). Mitochondrial calcium efflux occurs primarily through the mitochondrial Na^+^/Ca^2+^-exchanger (mNCX), and although Na^+^-independent means of mitochondrial calcium efflux are more prominent in other tissues (e.g., kidneys and liver), their activity has been found to be relatively low in the heart (Carafoli et al., 1974; Crompton et al., 1977).

## hiPSC technology: Cardiomyocyte differentiation

hiPSC differentiation to cardiomyocytes draws upon insights from human embryonic development. hiPSC-CM differentiation relies upon generation of cardiac mesoderm and, ultimately, cardiomyocytes, through recapitulating the stage-specific release of signaling factors. To date, three main methodological approaches have shown success in generating hiPSC-derived cardiomyocytes in the laboratory: Embryoid body formation, inductive co-culture, and adherent monolayer culture. Here, we will review monolayer culture, which is the predominant method for differentiation.

In-depth characterization of signaling components regulating cardiac development, including fibroblast growth factors (FGF), Wnt, Nodal/Activin A and bone morphogenic proteins (BMPs), served as the foundation for directed differentiation (Yang et al., 2008). It was then possible to reenact the sequence of events leading to CM specification *in vitro* through the fine-tuned application of growth factors and small molecules that possess the capacity to modulate cardiac signaling. To date, Activin A, BMP2/4, and Wnt regulators (IWP-2, CHIR99021) have been identified as the most essential auxiliary components for optimal differentiation of hiPSC-CMs (Burridge et al., 2011; Lian et al., 2012).

## Maturation of Ca^2+^ handling in hiPSC cardiomyocytes

**FIGURE 2:**
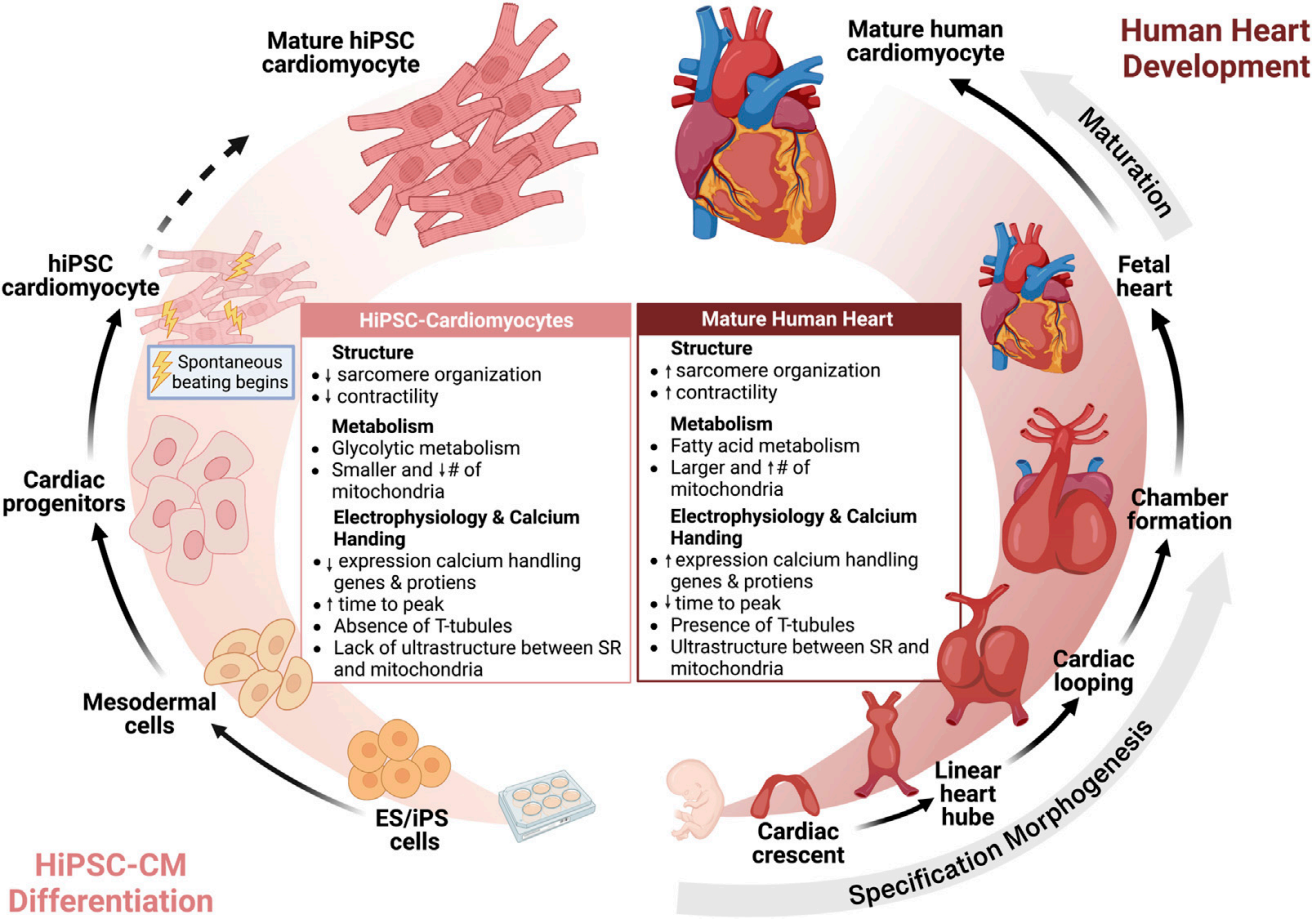
Human induced pluripotent stem cell cardiomyocytes are immature but reflect human heart development. Insights from cardiac morphogenesis has facilitated differentiation of hiPSC to hiPSC-CMs. Currently, hiPSC-CMs are immature compared to adult human cardiomyocytes, but further strategies for maturation may advance the maturity of hiPSC-CMs towards an adult phenotype.

The increased utilization of hiPSC-CMs has also renewed the interest in understanding cardiac development. During heart development, cells undergo specification, morphogenesis, and lastly maturation to form an adult heart. Maturation is defined by a developmental program that leads to the conversion of cardiomyocyte gene expression, function, cell structure, and metabolism from an immature fetal to mature adult cardiomyocyte (Karbassi et al., 2020) (Figure 2). While hiPSC-CMs have revolutionized the study of cardiovascular physiology and disease modeling, the well-acknowledged limitation of hiPSC-CMs is their relative immaturity compared to adult cardiomyocytes. hiPSC-CMs most closely resemble immature fetal cardiomyocytes which are characterized by less sarcomeric organization, spontaneous beating, reduced contractility, and dependence on glycolytic metabolism (Guo and Pu, 2020). With regards to calcium handling, hiPSC-CMs have express lower levels of calcium handling genes and proteins, increased time to peak, slower calcium signal decay, less well-developed calcium handling, absence of T-tubules, and lack of ultrastructural development and relationship between the SR and mitochondria, resulting in reduced coordination of cell-wide calcium (Zhang and Morad, 2016). In contrast, mature cardiomyocytes have higher expression of SERCA2a, RyR2, and other calcium handling genes and proteins, mature calcium regulation, T-tubule formation, organized SR, and low automaticity (Hwang et al., 2015; Zhang and Morad, 2016; Guo and Pu, 2020).

Thus, the cardiovascular stem cell field is highly invested in harnessing our understanding of cardiovascular development to drive maturity enhanced maturity, and importantly more robust Ca^2+^ handling in hiPSC-CMs. To this end, Ca^2+^ transient parameters, assessed by real-time detection of fluorescent Ca^2+^ dyes and indicators, are often used as positive markers for this improved cellular physiology.

### Long-term culture for maturation

**FIGURE 3:**
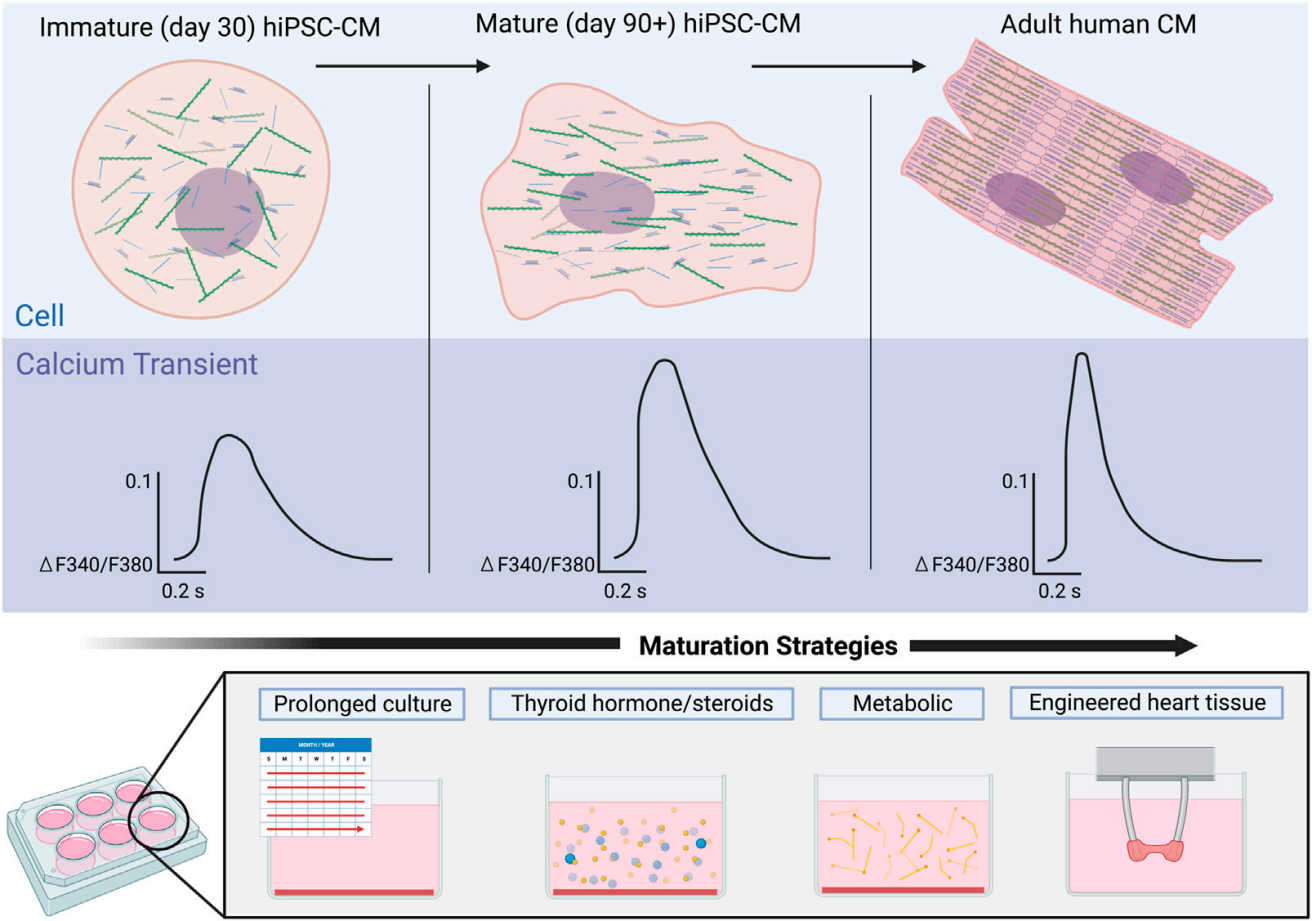
Maturation of calcium signaling in hiPSC-CMs over time in comparison to adult human cardiomyocytes. Early hiPSC-CMs (left) lack the structural organization of adult CMs (right), an immaturity reflected in their respective Ca^2+^ transients. Ca^2+^ transients of immature hiPSC-CMs exhibit a smaller amplitude as well as much slower rise times and recovery rates. Maturation of the hiPSC-CMs (middle) results in increased structural organization of the cells and Ca^2+^ transients with larger amplitudes and faster rise times and recovery rates. Strategies to mature Ca^2+^ handling in hiPSC-CMs will be critical to achieve more adult human like Ca^2+^ transients.

The initial approach to maturation of hiPSC-CMs was to increase the duration of time from differentiation to mimic temporal maturation of the human cardiomyocyte (Snir et al., 2003; Kamakura et al., 2013; Lundy et al., 2013). Characterization of hiPSC-CM differentiated and maintained for 1 and up to 4 months has shown an increase in maturation including sarcomeric organization, cell size and shape, and improved calcium amplitude and faster decay (Lundy et al., 2013; Pioner et al., 2019) (Figure 3). While hiPSC-CMs have been cultured for up to 1 year and have demonstrated an in sarcomeric organization, some aspects of maturation such as development of T-tubules have not be achieved with long term culture (Snir et al., 2003; Kamakura et al., 2013; Lundy et al., 2013). Some challenges of these early studies were the utilization of a single hiPSC line, variable differentiation protocols, and limited quantitative calcium imaging.

While there is maturation of hiPSC-CMs with long-term culture, the long-term culture is time-consuming and impractical for routine downstream experiments, thus to address these limitations the field has harnessed other aspects of cardiovascular development to drive the maturity of hiPSC-CMs in a shorter duration. Here we review maturation strategies that have been employed to mature Ca^2+^ handling including metabolic, hormonal, electrical, and engineered heart tissues (Table 1).

**TABLE 1:** Impact of maturation strategies on hiPSC calcium cycling.

Maturation methods	Effects on calcium/calcium transients	Reference
Fatty Acids - Metabolic	Increase in peak amplitude, upstroke velocity, recovery rate	Yang et al. (2019)
Biomimetic Substrate	Increase in peak amplitude, upstroke velocity, recovery rate	Pioner et al. (2019)
T3, Dex - Hormonal	Increase in upstroke velocity, recovery rate	Parikh et al. (2017)
T3, Dex - Hormonal	Increase in peak amplitude, upstroke velocity, & consistency of calcium transients throughout the cells (i.e., at the center vs. at the periphery)	Yang et al. (2014b)
Electrical, 3D culture	Increase in response to caffeine stimulation	Nunes et al. (2013)
Electrical Stimulation	Increase in peak amplitude, recovery rate. Decrease in rate of abnormal transients	Ronaldson-Bouchard et al. (2018)
3D Culture/EHT	Increase in peak amplitude	Shadrin et al. (2017)
3D Culture/EHT	Increase in peak amplitude, upstroke velocity, recovery rate	Kupfer et al. (2020)
Time/Long-Term Culture	Increase in peak amplitude, upstroke velocity, recovery rate	Lundy et al. (2013)

#### Metabolic maturation

A common strategy to drive maturity is through metabolic regulation, with supplementation of culture media or manipulation of metabolic signaling pathways. As adult cardiomyocytes rely predominantly on long-chain fatty acids to meet metabolic demand, their addition to culture media was theorized to yield a more mature energetic environment and enable improved Ca^2+^ handling. Indeed, the addition of palmitate, oleate and lineolate increases Ca^2+^ transient amplitudes and kinetics, which is consistent with the observation that inhibition of HIF-1α, and subsequent increase in fatty acid oxidation, enhance Ca^2+^ transient parameters and rates of cellular contraction and relaxation (Gentillon et al., 2019). However, the use of high glucose media also increases Ca^2+^ transient amplitudes and shortened decays (Balistreri et al., 2017), which may suggest that standard hiPSC-CM culture media may need to be adapted to provide the appropriate energetic fuel to maximize calcium handling maturity.

#### Biochemical maturation

Thyroid and glucocorticoid hormones are essential for normal *in vivo* cardiac development (Kruger et al., 2008; Lee et al., 2010; Rog-Zielinska et al., 2014; Rog-Zielinska et al., 2015). Tri-iodo-L-thyronine (T3) is the active form of thyroid hormone and is a strong transcriptional regulator of cardiac specific genes, including several involved in Ca^2+^ handling (SERCA2a, PLB, and NCX) (Chattergoon et al., 2012; Rog-Zielinska et al., 2013; Li et al., 2014), and supplementation has been found to enhance cardiomyocyte maturity (Yang et al., 2014b; Birket et al., 2015; Rog-Zielinska et al., 2015). Specifically, Yang et al. (2014b) showed that T3 treatment for 1 week, 3 weeks post-differentiation, resulted in greater contractile force, along with faster contraction and relaxation rates (Yang et al., 2014b). While T3-treated cells had comparable Ca^2+^ transient amplitudes to control cells, they showed much faster upstroke velocity and decay rates, with transients much more closely resembling those of adult cardiomyocytes. T3-treated cells also showed large increases in a-MHC and SERCA2a expression, and a significant increase in cell area and sarcomere length, as well as a decrease in cell circularity. T3 treatment also resulted in improvements in mitochondrial function, with oxygen consumption rates consistently higher than control cells in all instances.

In addition to thyroid hormones, glucocorticoids similarly regulate critical genetic programming necessary for development *in vivo* (Rog-Zielinska et al., 2013; Rog-Zielinska et al., 2014; Rog-Zielinska et al., 2015). In an experimental setting, treatment with T3 together with the synthetic glucocorticoid dexamethasone (Dex) further improves the functional maturity of hiPSC-CMs beyond T3 treatment alone (Yang et al., 2014b) (Parikh et al., 2017). Treatment with T3+Dex treatment resulted in a significant increase in T-tubule density in hiPSC-CMs, and improved intracellular Ca^2+^ handling, with Ca^2+^ transients having larger amplitudes and faster rise times. The Ca^2+^ propagation of treated cells was also much more consistent across cells, with similar Ca^2+^ transients in the center of the cell compared to in the periphery, a characteristic that is distinctly lacking in control cells. This improved coordination is consistent with improved ultrastructural development of cell-wide SR and mitochondrial networks. Ca^2+^ imaging, before and after blocking the release of SR Ca^2+^ with thapsigargin and ryanodine, showed that the release of SR Ca^2+^ contributes to a much larger amount of the cytosolic Ca^2+^ transient in the hiPSC-CMs treated with T3+Dex, again indicating a more developed SR structure. However, the T-tubules were not comparable in abundance or organization to adult cardiomyocytes.

#### Electrical and mechanical maturation

The heart is a dynamic tissue that undergoes continuous electrical depolarization and mechanical strain on a beat-to-beat basis, which has a tremendous impact on cellular structure and gene regulation during development and progression to an adult phenotype. These factors are then likely essential to fully drive hiPSC-CM maturity (Yang et al., 2014a). While standard differentiation protocols do produce cell-to-cell contacts (monolayers and EBs) that facilitate electrical conduction, they are significantly slower compared to the adult heart. hiPSC-CMs are also effectively unloaded, experiencing minimal cell stretch and physiological strain-induced genetic regulation (Lasher et al., 2012; Ruan et al., 2015; Veerman et al., 2015). Thus, several research groups have explored the application of mechanical stress and electrical stimulation, mimicking a more *in vivo* cellular environment of an adult cardiomyocyte. Such conditions do, in fact, improve Ca^2+^ transient kinetics (Kroll et al., 2017), and in some cases directly related to an increase in SERCA2a and RyR2 expression (Ruan et al., 2016). Spontaneous beating, a hallmark of immaturity, is thought to be related to the low expression of potassium inward rectifier channels, which stabilize the membrane potential (Barbuti et al., 2016). Therefore, increasing the expression of the potassium inward rectifier channel in hiPSC-CMs resulted in more mature hiPSC-CMs that had a loss of spontaneous beating, stable resting membrane potential and increased Ca^2+^ transient amplitudes (Vaidyanathan et al., 2016).

Biophysical stimulation through electrical pacing has been used to enhance the maturation of hESC-CMs and hiPSC-CM (Chan et al., 2013; Nunes et al., 2013; Kroll et al., 2017). Cells that received electrical stimulation developed a higher percentage of rod-like cells, sarcomeric alignment, more mature electrophysiology parameters, and enhanced Ca^2+^ transients (Chan et al., 2013; Nunes et al., 2013; Kroll et al., 2017).

In another study, to assess the effects of electrical stimulation on hiPSC-CM maturity (Ronaldson-Bouchard et al., 2018), cells were divided into three treatment groups: control (no stimulation), constant (2 Hz for 3 weeks) and intensity training (frequency increasing from 2 Hz to 6 Hz by 0.33 Hz per day, followed by a final week at 2 Hz). After constant stimulation, cells showed greater contractile force across a wide range of extracellular Ca^2+^ concentrations compared to controls, which increased further in the intensity training group. This difference in contractile force was much more pronounced in cells that underwent electrical stimulation immediately post-differentiation than in CMs that were cultured for 4 weeks prior to electrical stimulation. Ca^2+^ transients of cells in the intensity training group were much more consistent than in control cells, with larger peak amplitudes and shorter durations. Both the constant and intensity training stimulation resulted in hiPSC-CMs exhibiting a positive force-frequency relationship (FFR), while control cells continued to display a negative FFR (Ronaldson-Bouchard et al., 2018).

#### Engineered heart tissue

Engineered heart tissues and cardiac organoids contain hiPSC-CM and scaffold or extracellular matrix, and possibly other cells in the heart that better recapitulate aspects of the human heart (Weinberger et al., 2017). Zhang D. et al.( 2013) demonstrated that *SERCA2A* and *CASQ2* genes increased significantly in 3D fibrin-based cardiac patch cultures of human embryonic stem cells compared to 2D human embryonic stem cell derived cardiomyocytes (Zhang D. et al., 2013). Cardiomyocytes are thought to mature in the presence of multiple cell types in cardiac organoids. Therefore, Richards et al. developed scaffold-free hiPSC organoids and included human ventricular cardiac fibroblasts and human umbilical vein endothelial cells and demonstrated that these organoids have functional calcium handling in response to verapamil, a calcium channel blocker, ryanodine, and isoproterenol (Richards et al., 2017). Similarly, cardiac patches that were generated by seeding a mixture of cells (86% hiPSC-CMs, the remaining 14% made up of smooth muscle cells and fibroblasts) in hydrogel squares (Shadrin et al., 2017). Following 3 weeks in culture, the cardiac patches consisted of densely packed multilayered hiPSC-CMs that exhibited organized striations and gap junctions surrounded by smooth muscle cells and fibroblasts. During those 3 weeks, hiPSC-CMs consistently showed increased levels of expression of genes indicating structural maturity (TNNI3, MYL2, MYOM2, MYOM3) and metabolic maturity (COX6A2, CKMT2, CKM), as well as E-C coupling (CASQ2, S100A1, PLN). Ca^2+^ imaging showed an increase in Ca^2+^ transient amplitudes as the 3D culture time also increased. Upon implantation in the rat epicardia, these cardiac patches displayed a conduction velocity comparable to that of the rat heart tissue pre-implantation and did not interrupt electrical propagation across the heart (Shadrin et al., 2017).

Kupfer et al.(2020) utilized bioprinting with a specialized bio-ink and undifferentiated hiPSC with *in situ* cardiac differentiation to generate a chambered human muscle pump. They demonstrated that calcium transients were similar at 2 weeks and 6 weeks and had an expected response to isoproterenol (Kupfer et al., 2020). Further advances in cardiac tissue engineering and comparison to 2D hiPSC-CM will be important for head to head comparisons of cardiac calcium handling maturation.

#### Outlook

The wide variations in differentiation and maturation protocols yield a hiPSC-CM quality that differs significantly between studies. However, cells generally have sufficient Ca^2+^ cycling machinery to study Ca^2+^ transient defects in numerous inherited conditions (Kamdar et al., 2015) such as Brugada syndrome (Liang et al., 2016), catecholaminergic polymorphic ventricular tachycardia (Fatima et al., 2011), Friedreich ataxia (Crombie et al., 2017), and Duchenne muscular dystrophy (DMD) cardiomyopathy (Kamdar et al., 2020), in addition to several others detailed later in this review (Table 2). CRISPR/Cas9 has been used to correct DMD-causing dystrophin mutations in hiPSC lines, restore Ca^2+^ transient parameters, and reduced arrhythmic events (Kyrychenko et al., 2017). Limb-girdle muscular dystrophy arrhythmias can be recapitulated in patient-derived hiPSC-CM (30–60 days) associated with reduced Cav1.2 expression and conductance (El-Battrawy et al., 2018). Pathological phenotypes with no known molecular mechanisms are also excellent candidates for study by hiPSC-CM, including stress cardiomyopathy, which were observed to have enhanced β-adrenergic responsiveness and toxicity. Furthermore, hiPSC-CMs, despite their immature phenotype, have been used to recapitulate ischemia/reperfusion injury (Wei et al., 2017; Hakli et al., 2021). Although hiPSC-CMs are a revolutionary tool for studying cardiovascular diseases, the goal remains to mature hiPSC-CM towards an adult phenotype to enhance our knowledge of human cardiac physiology and pathophysiology.

**TABLE 2:** Summary of hiPSC calcium changes in cardiovascular disease modeling.

Disease Modeled	Gene Affected and Specific Mutations	Effects on Calcium/Calcium Transients	Reference
PLN DCM	PLB (R9C)	Shortened calcium transient decay, shortened relaxation time, blunted adrenergic response	Ceholski et al. (2018)
PLN DCM	PLB (R14 Deletion)	Irregular calcium transients, corrected by gene editing	Karakikes et al. (2015b)
CPVT1	RyR2 (F2483I)	Increase in peak amplitude, decrease in recovery rate	Fatima et al. (2011)
CPVT1	RyR2 (F2483I)	Decrease in peak amplitude, upstroke velocity, recovery rate	Zhang et al. (2013b)
CPVT1	RyR2 (R420Q)	Decrease in peak amplitude	Novak et al. (2015)
CPVT2	CASQ2 (D307H)	Decrease in peak amplitude and upstroke velocity, increase in diastolic calcium levels	Novak et al. (2015)
cTnT DCM	TNNT2 (K210 Deletion) TNNT2 (E160 Deletion)	Decrease in peak amplitude	Li et al. (2021b)
cTnT DCM	cTnT (R173W)	Reduced calcium transients, rescued by SERCA overexpression	Sun et al. (2012)
HCM	MYH7 (A663H)	Increased irregular calcium transients, increased diastolic calcium, rescue with verapamil	Lan et al. (2013)
HCM	MYL2 (R58Q)	Decrease in peak amplitude and recovery rate, increase in arrhythmic calcium transients	Zhou et al. (2019)
HCM	MYH7 (R663H)	Decrease in peak amplitude, increase in diastolic calcium levels and rate of abnormal calcium transients	Wu et al. (2019)
	MYBPC3 (V321M)		
	MYBPC3 (V219L)		
	TNNT2 (R92W)		
HCM	MYH7 (Arg442Gly)	Decrease in peak amplitude, increase in diastolic calcium levels	Han et al. (2014)
Timothy Syndrome/LQT8	CACNA1C	Increase in peak amplitude, decrease in recovery rate and frequency of spontaneous beating	Yazawa et al. (2011)
DMD	Dystrophin (exon 4–43, 3–7)	Increase in arrhythmic calcium transients, rescue with beta blockers	Kamdar et al. (2020)
DMD	miRNA-378a Deletion	Increase in irregularity of spontaneous beating frequency	Martyniak et al. (2021)
DMD	Dystrophin	Decrease in peak amplitude, upstroke velocity, recovery rate, increase in diastolic calcium levels	Chang et al. (2021)

### Ca^2+^ handling proteins in hiPSC-CM and implications for disease modeling

To assess the utility of hiPSC-CM for cardiovascular disease modeling, it is critical to assess and understand the differences in calcium regulation between hiPSC-CMs and the adult human heart. Overall, hiPSC-CMs express the major components involved in Ca^2+^ handling including SERCA2a, PLB, RYR2, and calsequestrin, though hiPSC-CM Ca^2+^ transients are less mature than adult cardiomyocytes (Germanguz et al., 2011; Itzhaki et al., 2011; Lee et al., 2011; Rao et al., 2013). In this section, we review the differences in calcium regulation between hiPSC-CM and adult cardiomyocytes by calcium transport proteins and relevant disease models.

#### SERCA/PLB

SERCA2a and PLB are expressed in hiPSC-CMs, though at lower levels than in hESC-CMs and adult cardiomyocytes (Itzhaki et al., 2011; Lee et al., 2011). In contrast to adult cardiomyocytes, hiPSC-CMs are dependent on both sarcolemmal Ca^2+^ transport and intracellular Ca^2+^ store release, which is comparable to fetal and post-natal cardiomyocyte and attributed to a less developed SR (Lee et al., 2011). While SERCA2a is expressed in hiPSC-CM, it was critical to assess whether SERCA2a was functional in hiPSC-CMs. Thapsigargin, a SERCA2a inhibitor, was applied to hiPSC-CMs to determine the contribution of SERCA2a to the Ca^2^ transient. 1–10 µM thapsigargin decreased the Ca^2^ transient amplitude in a dose-dependent manner, and 20 µM thapsigargin ablated the Ca^2^ transient (Itzhaki et al., 2011). Moreover, to determine if thapsigargin inhibition resulted from a decrease in the SR Ca^2^ load due to SERCA2a uptake inhibition, caffeine was applied to hiPSC-CM in the presence of thapsigargin. In hiPSC-CMs treated with thapsigargin, there was a minimal effect of caffeine, which demonstrates the inability of the SR to reload due to thapsigargin inhibiting SERCA (Itzhaki et al., 2011).

Furthermore, increased Ca^2+^ transient amplitude, shortened decay, and increased SR Ca^2+^ load (all attributable to increased SERCA activity) have been used as markers to assess improved differentiation maturity (Lee et al., 2011; Ng et al., 2011; Streckfuss-Bomeke et al., 2013) even when SERCA expression is not directly assessed. A two-fold or more change can be observed in these parameters, indicating a wide range in developmental variation of SERCA activity.

Beyond modification of pharmacological protocols and increased differentiation time, driving structural development through mechanical approaches greatly improves SR Ca^2+^ handling. Culturing cells on a micropatterned surface to generate cellular alignment increases Ca^2+^ transient amplitude and decay indicative of increased SERCA activity (Khan et al., 2015). Similar Ca^2+^ transient effects are observed in engineered tissue (Gao et al., 2017), suggesting that improved cellular organization is critical for the development of Ca^2+^ transport, likely through improved SR membrane structure and content.

PLB is a critical downstream target of adrenergic stimulation, where PKA and CaMKII can phosphorylate PLB, thereby tapping an activity reserve capacity of SERCA. Consistent with this, low PLB expression in immature cells contributes to a blunted ionotropic response to *β*-agonist (Chen et al., 2015), and in some early studies, a complete lack of PLB is observed (Dolnikov et al., 2006). In numerous other studies where PLB is not directly quantified, a faster Ca^2+^ transient decay is observed in response to isoproterenol, probably associated with increased phosphorylation of PLB (Cao et al., 2012; Lian et al., 2012; Correia et al., 2014; Hayakawa et al., 2014; Song et al., 2015). However, frequently in these studies, relative maturity is difficult to discern, as days post-differentiation are not indicated. PLB expression has been observed to increase over time in cultured cells (Lian et al., 2012; Shadrin et al., 2017; Cai et al., 2019) and through incorporation into engineered tissue (Cai et al., 2019; Murphy et al., 2019), likely contributing to enhanced adrenergic responsiveness.

Although SERCA2a and PLB are not major loci for disease causing mutations, a few mutations have been identified with PLB, with the disease phenotype extending to mutant hiPSC cell lines. In engineered hiPSC cardiac tissue, the PLB R9C mutation causes a shortened Ca^2+^ transient decay in cells and shortened relaxation time along with a blunted adrenergic response (Ceholski et al., 2018; Stroik et al., 2020). Patient-derived hiPSC-CMs from a carrier of PLN R14 deletion mutation exhibit irregular Ca^2+^ transients associated with higher beating rate and occurrences of Ca^2+^ waves (Karakikes et al., 2015b).

A key molecular dysfunction identified in human dilated cardiomyopathy and arrhythmogenesis involves the transport of calcium needed for the relaxation of cardiomyocytes in diastole, which is usually associated with the impaired activity of SERCA2a (Hasenfuss et al., 1994; Schwinger et al., 1995; Zarain-Herzberg et al., 1996; Davia et al., 1997; Tweedie et al., 2000; Haghighi et al., 2006). Structure-function studies of human SERCA2a overexpressed in human embryonic kidney cells indicate that large-scale movements of SERCA2a′s domains couple ATP hydrolysis to Ca transport (Moller et al., 2010). In cardiomyopathy, deficient Ca^2+^ removal from the cytoplasm is associated with decreased SERCA2a activity and an increased ratio of PLB to SERCA2a (MacLennan and Kranias, 2003). SERCA2a gene therapy in preclinical models and initial cardiomyopathy was successful in improving heart failure and arrhythmias, definitely showing that SERCA2a activation is a promising approach to treating heart failure. However, larger clinical trials of SERCA2a gene therapy failed, primarily due to the difficulty of delivering the large SERCA2a gene to patients on a large scale (Zsebo et al., 2014; Greenberg et al., 2016).

Fluorescent spectroscopic probes have been developed to understand the structural dynamics and function of SERCA2a and PLB (Schaaf et al., 2017a; Schaaf et al., 2017b; Lo et al., 2017; Schaaf et al., 2018). Fluorescent SERCA2a biosensors have been used in HEK293 cells to create a living cell biosensor. Time-resolved fluorescence resonance energy transfer (FRET) allows for detection of structural changes and activity in SERCA2a in living cells (Robia et al., 2007; Muretta et al., 2010; Gruber et al., 2012; Hou et al., 2012; Cornea et al., 2013; Pallikkuth et al., 2013; Gruber et al., 2014b; Schaaf et al., 2017a; Schaaf et al., 2018). SERCA2a activity is determined by the conformational state of SERCA2a, which can be detected by measuring the intramolecular distance between the acceptor and donor fluorescent proteins domains located on the cytoplasmic aspect (Cornea et al., 2013; Gruber et al., 2014a; Schaaf et al., 2017a; Schaaf et al., 2018). Prospective pharmacologic agents can be added to determine the impact of these agents on SERCA2a activity. These FRET biosensors have been limited to non-cardiac HEK293 cells, which has limited the utility of therapeutic discovery and understanding disease states, as the key structural and functional cardiac proteins needed for cardiac excitation and coupling are not expressed. The application of this technology in hiPSC-CM is currently being implemented and can allow functional and physiological measurements of calcium handling in disease models, high-throughput drug screening to identify allosteric SERCA2a activators, and drug toxicity assessment.

#### RyR2

RyR2 has been observed to increase over time and thus, has been used as a marker of hiPSC-CM quality for cells (Cao et al., 2012) and engineered tissue (Gao et al., 2017), indicating maturation towards adult like Ca^2+^ handling. Caffeine releases the SR calcium stores that are gated by RyR, and in hiPSC-CMs caffeine-induced Ca^2+^ store release was similar to those seen in adult small animal cardiomyocytes (Hwang et al., 2015). Application of ryanodine, a RyR2 inhibitor, reduced the amplitude, maximal upstroke, and decay velocity of hiPSC-CM Ca^2+^ transientsCa^2+^ release in hiPSC-CMs (Lee et al., 2011; Ng et al., 2011; Zhang and Morad, 2016). These data suggest that hiPSC-CMs have functional SR Ca^2+^ stores, but may have immature mechanisms of release.

RyR2 is a well established locus for arrhythmogenic mutations, through perturbation of Ca^2+^ gating and aberrant Ca^2+^ release. Gain-of-function pathogenic RyR2 mutantations appear particularly well suited to being modeled in hiPSC-CM, commonly recapitulating basic patient phenotypes. Catecholaminergic polymorphic ventricular tachycardia type 1 (CPVT1) is known to arise from mutations to RyR2. CPVT1 is characterized by abnormal intracellular Ca^2+^ handling and in conjunction with increased catecholamine levels result in DADs and ventricular arrhythmias. It is unknown whether the arrhythmic activity in CPVT1 is due to SR Ca^2+^ leak following RyR2 phosphorylation or increased Ca^2+^ sensitivity leading to diastolic Ca^2+^ release. Numerous groups have utilized hiPSC-CM to study specific RyR2 mutations causing CPVT1, from cells directly collected from patients, or through gene editing to introduce known CPVT1 mutations.

hiPSC-CMs derived from patients with the *F2483I* variant in RyR2 exhibited diminished Ca^2+^ release upon exposure to caffeine, indicating smaller stores of Ca^2+^, as well as a similarly diminished I_NCX_ (Fatima et al., 2011; Zhang X. H. et al., 2013). However, the cells seemed to have compensated for their depleted Ca^2+^ stores by increasing the fractional release of Ca^2+^ resulting from calcium induced calcium release during spontaneous beating. This was accompanied by a significantly longer duration of Ca^2+^ transients, consistent with abnormalities in SR Ca^2+^ release in cells with RyR2 mutations that underlie CPVT1.The *F24831* variant hiPSC-CMs also showed longer and farther-reaching Ca^2+^ sparks capable of activating nearby release sites with Ca^2+^ overload. F2483I variant gene edited hiPSC-CMs also exhibited longer and wandering Ca^2+^ sparks, elevated diastolic Ca^2+^ leak, and smaller SR Ca^2+^ stores, similar to that of F2483I patient-derived cells (Wei et al., 2018).

To understand how CPVT1 mutations in different domains of RYR2 inpact Ca^2+^ handling, hiPSC were gene edited to have *RYR2* mutations in the N-terminal (*R420Q)*, C-terminal (*Q4201R*), and central-domain (*F42831)*. hiPSC-CMs from all three *RYR2* variant lines had wandering sparks with abberrant focal Ca^2+^ release. The magnitude of Ca^2+^ leak was larger and SR content was larger in the *F2483I* and *Q4201R* variants than the *R420Q* mutant hiPSC-CMs. The increase in spark frequency and large leaks in the *F2783I* could be due to its position near the FK506-binding protein, that results in dissociation of the FK506-binding protein from RyR2 to result in larger leaks. The location of the mutation impacts the calcium induced calcium release gain, SR Ca^2+^ content, leakiness, and resposne to arrhythmic medications (Zhang et al., 2021). The *S406L* variant resulted in hiPSC-CMs that exhibited similar Ca^2+^ activity to control cells under resting conditions. However, isoproterenol stimulation of these mutant hiPSC-CMs caused elevated diastolic Ca^2+^ levels and Ca^2+^ spark frequency, resulting in an increase in DADs (Jung et al., 2012).

Investigation of other hiPSC-CMs with a variety of pathogenic *RYR2* mutations have alson demonstrated an increase in DADs, arrhythmias, SR Ca^2+^ leak, and increased RyR2 phosporylation (Itzhaki et al., 2012; Di Pasquale et al., 2013; Paavola et al., 2016; Preininger et al., 2016; Acimovic et al., 2018; Mehta et al., 2018; Wei et al., 2018; Polonen et al., 2020; Word et al., 2021). Thus, evaluating different RYR2 mutations can lead to mechanistic molecular understanding of disease progression, and provide a platform for precision medicine.

#### Plasma membrane transport (LTCC)

Sarcolemmal Ca^2+^ transport represents an important aspect of Ca^2+^ regulation and excitation-coupling, including for hiPSC-CMs. Application of nifedipine, a L-type calcium channel blocker (LTCC) o hiPSC-CMs led to the elimination of the whole cell Ca^2^+ transient, which suggests that Ca^2+^ entry *via* the LTCC is important for generation of the whole cell transient in hiPSC-CMs (Itzhaki et al., 2011)

Long QT Syndrome 8 (LQT8), or Timothy Syndrome, is caused by a missense mutation in *CACNA1C*, which encodes the Cav1.2 component of the L-type Ca^2+^ channe and can lead to fatal-ventricular arrhythmias. Insights into the pathophysiology has been limtied due to the LQTS mouse having different electrical properties, however LQTS8 has been modeled using hiPSC-CMs (Huikuri et al., 2001).

Patient-derived LQT8 hiPSC-CMs spontaneously beat at half the frequency of wild-type hiPSC-CMs. They also exhibited delays in L-type Ca^2+^ current inactivation and alterations to intracellular Ca^2+^ handling as evidenced by Ca^2+^ transients with larger and more inconsistent amplitudes as well as longer durations. LQTS hiPSC-CM, also showed a prolonged action potential and DADs. The LQTS8 electrical and Ca^2+^ handling abnormalities could be ameliorated by roscovitine, a small molecule, that increases voltage-dependent inactivation of CaV1.2. (Yazawa et al., 2011).

LTCC current response to Mg increases over time, reflecting maturity similar to adult cardiomyocytes (Nguemo et al., 2014). As would be expected with adult cells, LTCC current inhibtion is arrythmogenic in hiPSC-CM (Wu et al., 2017). Pharmacological induction of ER stress also down-regulates Cav1.2, contributing to arrhythmogenesis *via* the unfolded protein response (Liu et al., 2018).

#### Calcium buffering

As outlined in prior sections, Ca^2+^ cycling has a critical role in regulation of excitation-contraction coupling in the cardiomyocyte (Bers, 2008; Eisner et al., 2017), and a major component of calcium regulation and homeostasis is Ca^2+^ buffering both in the SR and cytoplasm (Smith and Eisner, 2019). In this section, the role of the SR Ca^2+^ buffer calsequestrin (Csq2) and cytoplasmic Ca^2+^ buffer, namely cardiac troponin C, will be discussed including the impact of dysregulated Ca^2+^ buffering and insights from hiPSC-CM disease modeling.

##### Calsequestrin

CASQ2 encodes cardiac calsequestrin (Csq2), a high-capacity, low-affinity Ca^2+^ binding protein, that is the major cardiac SR Ca^2+^ buffer (Ikemoto et al., 1972; Yano and Zarain-Herzberg, 1994). The ability of Csq2 to buffer SR Ca^2+^ facilitates the repeated cardiomyocyte contraction necessary to support cardiac output. Thus, alterations in SR Ca^2+^ buffering *via* Csq2 have a significant impact on intracellular Ca^2+,^ as *CASQ2* mutations result in catacholaminergic polymorphic ventricular tachycardia 2 (CPVT2) (Ng et al., 2020). In both CPVT1 and CPVT2 there is abnormal Ca^2+^ from the SR, but in contrast in CPVT2 the mechanism is secondary to a loss of function mutation in *CASQ2.* CPVT2 clinically results in DADs and catecholamine triggered ventricular tachycardia, however the current guideline directed therapy, β-blockers, can fail to prevent fatal arrhythmias, thus there is a need to identify novel therapies (Priori et al., 2013).

Casq2 expression has been identified in hiPSC-CM and increase with time in cultured cells and through incorporation into engineered tissue, likely contributing to improved Ca^2+^ transient kinetics (Itzhaki et al., 2011; Gao et al., 2017; Cai et al., 2019). Patient-derived CPVT2 hiPSC-CMs with a CASQ2 D307H mutation, exhibited a decrease in the Ca^2+^ transient amplitude, an increase in diastolic Ca^2+^ levels, although they also showed a faster rise in Ca^2+^ transient. In control cells, caffeine exposure resulted in a sharp increase in diastolic Ca^2+^ levels, which returned to a steadystate within approximately 20 s. However, in the CPVT2-hiPSC-CMs, diastolic Ca^2+^ levels showed a much larger increase and remained elevated for much longer, only returning to a steady-state after 50–60 s (Novak et al., 2015). Similar results are also observed with other CPVT2 *CSQ2* mutations: the *CASQ2-G112+5X* mutation exhibits DADs in patient-derived hiPSC and can be corrected by non-mutant Csq2 overexpression (Lodola et al., 2016) and the D307H mutation in CSQ in patient-derived hiPSC causes EADs (Maizels et al., 2017).

#### Sarcomere proteins

Sarcomeric proteins, including cardiac troponin C and β-myosin heavy chain, play a critical role in cardiomyocyte contractility. Contraction is dependent on the interaction of the thin actin and the thick myosin filament, which is initiated by electrical activation of the cardiomyocyte and the resultant increase in intracellular Ca^2+^ (Huxley, 1957). During activation the majority of intracellular Ca^2+^ is bound to calcium buffers, including SERCA2a and troponin C (Smith and Eisner, 2019). Increasing buffering results in changes in a decreased Ca^2+^transient amplitude and the rate constant of Ca^2+^ decay (Diaz et al., 2001). Thus, while sarcomeric proteins do not participate directly in Ca^2+^ transport, they play a crucial role in cardiomyocyte Ca^2+^ homeostasis. β-adrenergic stimulation also results in sarcomeric protein phosphorylation *via* protein kinase and in combination with SERCA2a activation results in increased intracellular Ca^2+^ to promote inotropic, chronotropic, and lusitropic responses in the myocardium (Bers, 2002). The critical nature of sarcomeric proteins is revealed when patients have mutations in sarcomeric proteins that can lead to genetic dilated cardiomyopathy (DCM) and hypertrophic cardiomyopathy. Although patients with DCM and HCM have different phenotypes, a commonality of the phenotypes includes contractile defects, ventricular arrhythmias, and sudden cardiac death, all of which have aberrant Ca^2+^ cycling involvement (Kamisago et al., 2000).

DCM results in reduced contractile function leading to left ventricular dilatation, ventricular arrhythmias, and systolic heart failure. Genetic DCM accounts for over a third of non-ischemic cardiomyopathies (Hershberger et al., 2013). Genetic DCM is most commonly due to gene encoding sarcomeric proteins, including titin (*TTN)*, myosin heavy chain (*MYH7*), and cardiac troponin T mutations (*CNNT).* (McNally and Mestroni, 2017).

Several groups have modeled genetic and acquired DCM using hiPSC-CM to uncover molecular insights into the pathophysiology (Siu et al., 2012; Sun et al., 2012; Hinson et al., 2015; Wu et al., 2015; Yang et al., 2018; McDermott-Roe et al., 2019; Li B. et al., 2021). Consistent with the clinical manifestations, hiPSC-CMs with DCM mutations have been identified to have abnormal myocardial structure and dysregulated Ca^2+^ signaling. Cardiac troponin T, encoded by *TNNT2,* is a common DCM mutation that has altered Ca^2+^ handling and response to adrenergic signaling (McNally and Mestroni, 2017). Patient-derived hiPSC-CMs with a *TNNT2* R173W mutation were used to recapitulate the DCM phenotype and decreased contractility, reduced Ca^2+^ transient parameters consistent with reduced SR Ca^2+^ load, and abnormal α-actinin distribution, which could be corrected with the β-blocker metoprolol (Sun et al., 2012). Subsequently, the role of β-adrenergic signaling was further evaluated in the *TNNT2* R173W hiPSC-CMs, and a blunted β-adrenergic pathway was identified in these DCM hiPSC-CMs. Furthermore, they discovered that this mutation could promote nuclear localization of *TNNT2* and modify the epigenetic regulation of key β-adrenergic signaling genes (Wu et al., 2015). Cardiac troponin T mutations can result in both DCM and hypertrophic cardiomyopathy (HCM). Li et al.(2014) sought to compare two *TNNT2* mutations, K210, which causes DCM, and E160, which results in HCM through the generation of TALEN-mediated hESC lines with both heterozygous or homozygous mutations. Distinct phenotypes were identified between mutations. hiPSC-CMs with the heterozygous *TNNT2* K210 mutation exhibited many physical hallmarks of DCM such as fewer myofibrils, disrupted sarcomere organization, and punctate cardiac troponin T distribution. Furthermore, these hiPSC-CMshad abnormalCa^2+^ transients, with a more irregular frequency and shorter durations. In the homozygous *TNNT2* the structural flaws were much more pronounced in cells with the homozygousK210 mutation, and their Ca^2+^ transients had much smaller amplitudes. In contrast, hiPSC-CMs with the heterozygous E160 mutation had denser myofibrils, thicker Z-lines, and increased contractility consistent with HCM. Although all mutant hiPSC-CMs demonstrated irregular beating corresponding to arrhythmias, the *TNNT2* E160 hiPSC-CMs had a lower frequency and amplitude with slower rise times and longer recovery (Li B. et al., 2021).

In contrast to DCM, HCM is characterized by severe hypertrophy of the left ventricle leading to left ventricular outflow obstruction, heart failure, ventricular arrhythmias, and sudden cardiac death (Maron et al., 2022a; Maron et al., 2022b; Maron et al., 2022c). hiPSC-CMs have also been utilized to model a variety of HCM mutations. Patient-derived hiPSC-CM carrying a *MYH7* R663H mutation demonstrated cell enlargement, arrhythmias exacerbated by isoproterenol, and increased intracellular Ca^2+^ (Lan et al., 2013). Since calcium handling abnormalities appeared to precede the hypertrophic phenotype, it was hypothesized that calcium handling may be central to the phenotype. To test this hypothesis, HCM hiPSC-derived CMs were treated with calcium channel blockers verapamil, nifedipine, and diltiazem; and the results demonstrated prevention of myocyte hypertrophy, arrhythmias, and calcium handling abnormalities. This study demonstrated that aberrant calcium handling is a central mechanism in HCM (Lan et al., 2013).

In another study, hiPSC-CMs were generated from four HCM patient-derived hiPSC lines that including a variety of sarcomeric mutations including:*MYH7* R663H, *MYBPC3* V321M, *MYBPC3* V219L, and *TNNT2* R92W (Wu et al., 2019). All HCM hiPSC-CMs recapitulated diastolic dysfunction and arrhythmic phenotype, including diastolic Ca^2+^ overload, increased myofilament Ca^2+^ sensitivity, andshorter Ca^2+^ transient peaks while being paced. The HCM phenotype could further be exacerbated through β-adrenergic stimulation and partially rescued with the calcium channel blocker diltiazem, and nearly fully rescued with verapamil and the sodium channel inhibitor ranolazine (Wu et al., 2019).

Others have also modeled a variety of HCM mutations using hiPSC-CMs and have also recapitulated the HCM phenotype, including aberrant calcium regulation and arrhythmias. (Toepfer et al., 2019; Zhou et al., 2019; Toepfer et al., 2020; Hsieh et al., 2022). Further investigation of the pathophysiology of HCM using hiPSC-CM will be important to better understand the genotype-phenotype relationship, the impact of novel myosin inhibitors on calcium regulation, and identification of novel disease-specific therapies.

#### Cytoskeleton proteins

Cytoskeletal proteins play a critical role in cardiac maintenance of mechanical integrity, mechanotransduction, sarcomere stabilization, but also modulation of signaling pathways in the myocardium, and mutations in cytoskeletal genes can result in cardiomyopathy (Kostin et al., 2000). Duchenne muscular dystrophy (DMD) is characterized by the absence of the full-length membrane associated cytoskeletal protein, dystrophin, leading to severe muscle weakness, cardiomyopathy, and premature death (Kamdar and Garry, 2016). Ventricular myocytes from muscular dystrophy mice show an increase in stretch-related intracellular calcium overload and dysregulation in calcium handling (Yasuda et al., 2005; Williams and Allen, 2007). The mechanism of Ca^2+^ dysregulation in the absence of dystrophin may be multifactorial due to increased intracellular sarcolemmal tears and alterations in the function of key Ca^2+^ cycling proteins; however the precise mechanism is not yet known (Law et al., 2020). However, DMD hiPSC-CMs have been increasingly used to assess Ca^2+^ dysregulation in DMD cardiomyopathy. Patient-derived DMD hiPSC-CMs (Kamdar et al., 2020) were used to model DMD cardiomyopathy. The control hiPSC-CMs exhibited regular Ca^2+^ transients with consistent amplitudes at baseline. Treatment with isopropterenol increased both the frequency and the time to peak of Ca^2+^ transients, while subsequent addition of propranolol led to a sharp decrease in the frequency of Ca^2+^ transients. At baseline, DMD-hiPSC-CMs exhibited highly irregular Ca^2+^ transients, displaying high variability in cycle length, as well as high amount of arrhythmic activity, including double beats and DADs. Treatment with isoproterenol similarly led to an increase in Ca^2+^ transient frequency, along with a large increase in arrhythmic activity. Subsequent treatment with propranolol significantly decreased Ca^2+^ transient frequency and reduced arrhythmic activity to levels below those at baseline. Moreover, propranolol treatment prior to isoproterenol maintained arrhythmic activity similar to that of baseline. *DMD* null isogenic control lines (iDMD-hiPSC-CMs) exhibited similar arrhythmic Ca^2+^ activity and responses to isoproterenol and propranolol as seen in the DMD-hiPSC-CMs derived from reprogrammed patient cells (Kamdar et al., 2020). Similarly, Chang et al.(2021) identified that hiPSC-CMs from multiple DMD patient lines exhibited irregular calcium activity, including elevated diastolic calcium levels, decreased transient amplitude, and longer recovery rates. Following a period of moderate mechanical stress intended to mimic fibrotic stiffening of the heart, DMD-hiPSC-CMs notably exhibited weaker contractile force compared to control hiPSC-CMs given the same stress, however, no such discrepancy was observed in cells subjected to a milder stimulus. Moderately stressed DMD-hiPSC-CMs also showed decreases in contraction and relaxation velocities, as well as overall cell size (Chang et al., 2021). Additionally, the microRNA-378a has been found to be altered in the serum of DMD patients, and deletion in hiPSC-CM depresses Ca^2+^ transients (Martyniak et al., 2021). Further investigation of calcium dysregulation in DMD cardiomyopathy using DMD hiPSC-CMs, other model systems, and patient samples will help to elucidate the major mechanisms and potential treatments.

## Conclusion

In conclusion, our understanding of the physiological and pathological regulation of cardiac calcium has been largely based on the utilization of isolated animal model cardiac tissue or cardiomyocytes. However, the discovery of hiPSC has been revolutionary in science, and the subsequent differentiation to cardiomyocytes in the last decade has provided a new platform of renewable human cardiac cells to deepen our understanding of the mechanisms of cardiovascular physiology and disease and the screening for therapeutics. In particular, inherited cardiovascular disease, including genetic arrhythmias and cardiomyopathies, have become more accessible to research with hiPSC-CM. While the molecular mechanisms of cardiovascular diseases are different, aberrant calcium signaling is common to the majority of them. Here, we have reviewed the important differences in calcium regulation between adult human cardiomyocytes, especially in relation to cardiovascular disease modeling.

## Limitations of hiPSC-CM and need for maturation

Compared to adult human cardiomyocytes, hiPSC-CMs are less mature. While hiPSC-CMs are a promising translational tool for understanding cardiovascular disease, the maturity of hiPSC-CMs must be taken into account, especially their ability to regulate calcium, to model human cardiovascular disease. Although hiPSC-CM are relatively immature, they may be useful in understanding early stage and developmental changes in calcium regulation, and hiPSC-CMs have many qualitative calcium handling properties similar to those of human cardiomyocytes. The majority of our understanding of calcium regulation in the heart stems from studies done on animal cardiomyocytes or diseased human hearts, and this should also be taken into consideration when evaluating hiPSC-CM calcium regulation.

These differences in calcium handling, especially related to maturity, are important for the field overall to understand the limitations of current hiPSC-CM technology and subsequently develop more mature hiPSC-CM that better recapitulate the human myocardium that is needed for disease modeling, drug toxicity screening, and clinical therapeutic applications.

hiPSC-CMs are a unique tool, but in order for hiPSC-CM to be utilized to understand mechanisms of calcium handling at baseline the maturity of hiPSC-CM including the ability to differentiate mature chamber specific CM is needed. Additionally, establishing expected parameters for calcium regulation at different stages of differentiation can better characterize and standardize hiPSC-CMs to evaluate the impact of maturation strategies and ensure appropriate disease modeling.

## Future directions

There are still many gaps in the knowledge regarding calcium regulation in hiPSC-CMs. A number of questions remain especially in regards to the structure and function of various calcium handling components and the coupling of components needed for excitation contraction coupling in hiPSC-CM.

The combination of hiPSC-CMs with biosensors, such as fluorescence resonance energy transfer (FRET) biosensors for SERCA2a which have typically been utilized in non-cardiac cells, can inform the field about the structure and function of these calcium handling components at baseline and in disease states. Additionally, hiPSC-CMs that express FRET biosensors for SERCA2a can be used for high-throughput drug screening to identify novel therapeutics that could be used to increase SERCA2a activity, which is reduced in heart failure states (Schaaf et al., 2020). These hiPSC-CM containing SERCA2a FRET biosensors could also be used to test drug toxicity of new therapies on SERCA2A function, which may help better understand impact of new drugs on calcium regulation and excitation coupling in the heart.

Similarly, other biosensors can be utilized in hiPSC-CMs to understand the contribution of mitochondrial calcium in the context of cytosolic calcium at baseline and in disease states, which is not currently well established (Ernst et al., 2021).

Gene editing hiPSC-CMs with CRISPR/Cas9 and newer precise editing methods can be utilized for both basic investigation and translational development, by assessing the impact of mutations in various calcium handling genes and their regulators, systematically assessing novel therapies, and for precision medicine to determine if specific mutations result in a phenotype. Genetic engineering strategies have great potential to help elucidate mechanisms of early cardiac calcium regulation and is an exciting area for the field.
